# DEVELOPMENT OF A TRANSITIONAL CARE PROGRAM FOR FRAIL OLDER ADULTS BETWEEN HOSPITAL AND HOME

**DOI:** 10.1093/geroni/igac059.2123

**Published:** 2022-12-20

**Authors:** Ji Yeon Lee, Eunhee Cho, Sue Kim, Gwang Suk Kim, Kyung Hee Lee, Chang Oh Kim

**Affiliations:** Yonsei University, Seoul, Seoul-t'ukpyolsi, Republic of Korea; Yonsei University, Seoul, Seoul-t'ukpyolsi, Republic of Korea; Yonsei University, Seoul, Seoul-t'ukpyolsi, Republic of Korea; Yonsei University, Seoul, Seoul-t'ukpyolsi, Republic of Korea; Yonsei University, Seoul, Seoul-t'ukpyolsi, Republic of Korea; Yonsei University, Seoul, Seoul-t'ukpyolsi, Republic of Korea

## Abstract

Frail older adults particularly need transitional care between hospital and home due to physical function decline and psychological instability after discharge. This study aimed to develop a transitional care for frail older adults in Korea who are discharged home following hospitalization. The Returning Home (Rehome©) program was established through the three phases according to the Medical Research Council’s 2013 guidelines. 1) Identifying the evidence base phase included a systematic review of literature and needs assessments from interviews with frail older adults. The core intervention components (e.g., geriatric assessment, transitional care planning, home visits, phone follow-up, community service liaison, and family engagement) were determined. 2) At the phase of identifying theory, the transition theory was selected and modified to fit the target population in the context of the Korean healthcare system. 3) Phase three was for the modeling process and outcomes. Based on the result from phases 1 and 2, the Rehome program was developed considering clinically applicable strategies. The final Rehome program consisted of a comprehensive geriatric assessment at admission; structured discharge/transitional care planning (e.g., medication review, education for chronic disease management, emergencies, and geriatric syndromes, and community resource) at discharge; a home visit and six phone follow-up calls up to 12 weeks after discharge; and emotional support and engagement of the family during the entire period. The Rehome program showed good content validity. The Rehome as a frailty-focused transitional care program could improve the transition through implementing a tailored intervention that meets the care needs of these vulnerable populations.

